# Development of Criteria for a Positive Front-of-Package Food Labeling: The Israeli Case

**DOI:** 10.3390/nu12061875

**Published:** 2020-06-23

**Authors:** Michal Gillon-Keren, Vered Kaufman-Shriqui, Rebecca Goldsmith, Carmit Safra, Iris Shai, Gila Fayman, Elliot Berry, Amir Tirosh, Dror Dicker, Oren Froy, Eli Gordon, Anat Chavia Ben-Yosef, Lesley Nitsan, Hava Altman, Moran Blaychfeld-Magnazi, Ronit Endevelt

**Affiliations:** 1 Institute of Endocrinology and Diabetes, Schneider Children’s Medical Center, Petah Tikva 4920235, Israel; michalgk@gmail.com (M.G.-K.); gilafa@gmail.com (G.F.); 2 Faculty of Sciences, Kibbutzim College of Education Technology and the Arts, Tel Aviv 6250769, Israel; 3 Department of Nutrition Sciences, School of Health Sciences, Ariel University, Ariel 40700, Israel; veredks@ariel.ac.il; 4 Nutrition Division, Ministry of Health, Jerusalem 9101002, Israel; gorebecca@gmail.com (R.G.); carmit.safra@moh.gov.il (C.S.); lesley.nitsan@gmail.com (L.N.); havaalt1@gmail.com (H.A.); moran.magnazi@moh.gov.il (M.B.-M.); 5 Department of Public Health, Faculty of Health Sciences, Ben-Gurion University of the Negev, Beer-Sheva 8410501, Israel; irish@bgu.ac.il; 6 Israeli National Council of Diabetes, Ministry of Health, Tel Aviv 6744325, Israel; 7 Braun School of Public Health, Hebrew University-Hadassah Medical School, Jerusalem 9222507, Israel; elliotb@ekmd.huji.ac.il; 8 Division of Endocrinology, Diabetes and Metabolism, Sheba Medical Center, Tel Hashomer, Ramat Gan 5266202, Israel; Amir.Tirosh@sheba.health.gov.il; 9 Sackler Faculty of Medicine, Tel Aviv University, Tel Aviv 6997801, Israel; drord@clalit.org.il; 10 Internal Medicine D, Hasharon Hospital, Rabin Medical Center, Petah Tikva 4937211, Israel; 11 The Institute of Biochemistry, Food Science and Nutrition, The Robert H. Smith Faculty of Agriculture, Food and Environment, The Hebrew University of Jerusalem, Rehovot 7610001, Israel; oren.froy@mail.huji.ac.il; 12 Food Control Services, Ministry of Health, Tel Aviv 6473912, Israel; eli.gordon@moh.gov.il (E.G.); anat.chavia@moh.gov.il (A.C.B.-Y.); 13 School of Public Health, University of Haifa, Haifa 3498838, Israel

**Keywords:** food labeling, front-of-pack (FoP) label, food policy, healthy eating, nutrition, regulation

## Abstract

Efforts to shape the food environment are aimed at reducing diet-related co-morbidities. Front-of-package labeling (FOPL) may support the consumers to make an informed decision at the point of purchase and encourage industry to reformulate food products. The Israeli Ministry of Health (MOH) implemented a unique FOPL system, using two colors: A mandatory warning (red) label alongside a voluntary positive (green) label. An independent Scientific Committee, from academia, the healthcare system, and MOH was appointed to determine the core principles for the positive FOPL. The criteria were based on the Mediterranean diet principles, with adjustments to the Israeli dietary habits, focusing on the health advantages of the food and considering its processing level. The food products eligible for positive FOPL are foods in their natural form or with added spices or herbs, or those that underwent minimal processing, with no food additives. Based on population consumption data, 19.8% of food products were eligible for positive FOPL; of them, 54% were fruits and vegetables, 20% dairy, and 14% grains. An evaluation plan is needed to assess the degree of acceptance of the positive FOPL by the industry, retailers, and the public, and its impact on food consumption and on public health.

## 1. Introduction

Israel is facing an obesity epidemic, with concern regarding overweight and obesity among children and adolescents. Fifty-eight percent of the adult population is overweight or obese, and similarly, 18% of 7-year-old children. The obesity rate is almost two-fold higher in the lowest compared to the highest socioeconomic class (30.5% vs. 16.1%, respectively) [1]. The economic burden of obesity, overweight, and sugar consumption in Israel, in terms of direct and indirect costs, is estimated at US $173 million per year (0.35% of gross domestic product, GDP) [2]. According to the latest Organization for Economic Co-operation and Development (OECD) report, obesity and overweight in Israel reduce the life expectancy by 2.8 years, and the healthy life expectancy by 3.4 years, and will cause a loss of 2.7% in GDP and increase the healthcare costs by US $100 per person per year [3].

Two of the major dietary factors associated with an increased risk of obesity are inadequate intake of fruits, vegetables, and other unprocessed foods and increasing consumption of ultra-processed foods and beverages [4,5,6]. Ultra-processed foods are usually energy-dense products high in fat, salt, and/or sugar and low in essential nutrients [7,8]. Excessive consumption of ultra-processed food is related to non-communicable diseases (NCDs), such as type 2 diabetes, cardiovascular disease, stroke, and some cancers and to higher rates of mortality [5,9,10,11,12]. Analysis of the latest National Health and Nutrition (MABAT) surveys indicated that Israeli adolescents consume half of their total energy from ultra-processed foods, whereas adults consume 41% of their total energy from ultra-processed foods (unpublished data from MABAT surveys) [13,14].

To tackle the obesity challenge and change the local obesogenic environment, the Israeli Ministry of Health (MOH) promoted a comprehensive nutrition policy. One of the policy’s components was an introduction of front-of-package labeling (FOPL) [15], as recommended by the World Health Organization (WHO) [16].

FOPL provides visible nutrition information on packaged foods, it is easily identified, and can simplify the food selection at the point of purchase, facilitating the choice of healthy food [17,18,19,20,21]. FOPL has been introduced in many countries worldwide, using diverse approaches, mandatory or voluntary, led by governments or by the food industry, providing nutrient information or nutrition advice [17,18,19,22]. There are four main types of interpretive FOPL systems. There are endorsement logos, an example of which is the Nordic Keyhole, wherein foods are eligible for the symbol if category-specific criteria are met [23], and summary indicator systems, such as the French Nutri-Score, which ranks foods from dark green (healthiest) to dark red (least healthy) [24]. Another system is the nutrient-specific label such as the United Kingdom’s traffic light, which provides in one graphic, a color-coded indication (red, amber, and green) for individual nutrients [25]. The fourth system is the nutrient-specific warning labels, such as the Chilean black and white stop sign [26].

From the variety of FOPL systems, interpretive systems that provide evaluative judgements about the healthfulness or unhealthfulness of the product, and use of meaningful colors to signpost the relative healthfulness of foods, are more effective in guiding consumers to make healthier food choices [18,19]. Unfortunately, most FOPL systems use industry-established criteria rather than science-based criteria or according to national nutritional recommendations. As a result, a positive or a better-ranking FOPL can be applied to ultra-processed foods with poor nutritional quality [27,28].

The interpretive FOPL system chosen in Israel is unique, using two colors to represent a negative or a warning (red) label and a positive (green) label (Appendix A). While the warning FOPL is mandatory, the positive FOPL is voluntary [15]. The warning FOPL was adopted from the Chilean system, based on research indicating its effectiveness in a population of diverse socioeconomic status [29,30]. In 2018, the MOH passed the legislation for the warning FOPL and from 1 January 2020 any food that exceeds the predetermined threshold for sugar, sodium, and saturated fat (but not for total energy) must carry warning labels. A second stage, with stricter maximum thresholds, will come into effect in January 2021. Food can carry one, two, or three warning labels (or no label), according to its composition (Appendix B).

A significant benefit of the FOPL is its impact on the composition of processed food, as it may encourage reformulation actions by the industry. Along with the benefits expected from the warning FOPL for sugar, sodium, and saturated fat, namely reducing their contents in foods, there can be negative consequences such as using artificial sweeteners instead of sugar or reducing packaging size to avoid warning FOPL [29,30,31,32,33,34,35,36,37,38]. The absence of warning FOPL might be perceived by the public as an indication of a healthy food, especially if processed foods are advertised as “complying with the requirements of the MOH” or “without warning labels”. Therefore, the presence of positive FOPL along with negative ones, to guide consumers to a healthy eating choice, is essential. In the Israeli labeling model, food is labeled as “positive” on its own merits and not as opposed to the warning labeling, meaning that food products that are not marked as “negative” are not necessarily labeled as “positive”. The absence of warning labeling does not imply that the product is a “healthy product”.

Before the determination of the criteria for the positive FOPL, the Israeli MOH carried out consultations with international experts, some of whom had participated in the committees of the Keyhole and the Choices labels. In addition, the influence of the “EFSHARIBARI” (“Healthy is Possible”) label was examined. This specific, positive FOPL is permitted for loaves of bread (including pita bread and rolls) containing at least 80% of grain as whole grain, no more than 400 mg sodium, and no more than 250 kilocalories per 100 g This label, implemented in 2013, has been promoted by the MOH and gained recognition and cooperation from manufacturers as well as consumers.

Following those processes, it was decided to appoint a Scientific Committee, (herein “the committee”) whose role it was to determine the food profiling and the core principles for the positive FOPL. The committee was made up of leading nutrition and medical professionals from academia and the healthcare system and personnel of the Food Control Services and the Nutrition Division of the MOH. Several meetings of the committee members were held with representatives of the food industry in order to understand the challenges faced by the industry. However, the committee insisted that the industry would not be part of setting the criteria definitions so as to avoid conflicts of interest. All committee members signed a conflict of interest declaration to verify the absence of conflicts. In this way, the committee was independent and worked for the benefit of the public’s health [4,39,40].

This study aimed to describe the development process and considerations of the positive FOPL criteria and to analyze the expected proportion of food items eligible for positive FOPL.

## 2. Methods

### 2.1. The Rationale for Determining the Positive FOPL Criteria

Most of the work of the committee was carried out between August 2017 and July 2018, during which five meetings and many online consultations took place. The committee conducted a rapid literature review and examined several FOPL systems, which used favorable judgment, from different countries. These included: The Nordic Keyhole [23], France’s Nutri-Score [24], UK’s Traffic Light [25], Australia and New Zealand’s Health Star Rating system [41], and Singapore’s Healthier Choice [42]. Food labeling implemented by food companies, such as Choices, was evaluated as well [43,44]. The recommendations of the WHO and other health organizations concerning food labeling and the various criteria used for the development of nutrient profiling models were also taken into account [20,31,45,46,47,48,49,50]. Their acceptance by the public and their impact on consumers and industry were also examined [32,34,35,36,37,38,51,52,53,54,55,56].

The committee faced the dilemma of having to categorize food products considering the way a food is produced, the ways the food is eaten and integrated into daily menus, and not only based on its nutrient composition. Based on the experience from around the world and WHO recommendations, taking into account Israel’s unique needs, the committee set forth clear targets for positive FOPL. At each stage, the positive FOPL criteria were examined against the defined objectives. The criteria were determined based on several principles: Support of the recommendations of the Mediterranean diet with adjustment to the Israeli dietary habits and consideration of food processing levels and its natural composition.

#### 2.1.1. Support of the Recommendations of the Mediterranean Diet

The primary role of the positive FOPL is to support and promote healthy diets by helping the public choose food according to the newly issued nutritional guidelines. The new Israeli nutritional guidelines, developed by the Nutrition Division in the Public Health Services of the MOH, are based on the four sustainable dimensions of the Mediterranean diet: Health, environment, economy, and society and culture [57,58]. The Mediterranean diet is a dietary pattern associated with lower morbidity and mortality rates from the major NCDs [59,60]. It is based on plant foods, seasonal, local, fresh, and mainly unprocessed foods. The Mediterranean diet is rich in vegetables, fruits, whole grains, legumes, nuts, and olive oil. Milk and dairy products are consumed in moderation and so are eggs, fish, and poultry, with red meat consumed in a small amount [61].

#### 2.1.2. Adjustment to the Israeli Dietary Habits

The positive FOPL criteria were adapted to the typical dietary habits and consumption data in Israel, based on MABAT surveys and the Israeli food and nutrient database (BINAT) [13,14,62]. Factors such as the current variety of food products on the market and the way the food is eaten were also taken into account. Foods eligible for a positive FOPL included additional food items, beyond the classic foods included in the Mediterranean diet, for example, tahini (sesame paste), hummus (chickpeas paste), canola oil, and quinoa, to expand the variety available and to be in tune with characteristics of Israeli dietary habits. These types of foods correspond to the basic Mediterranean diet principles and can provide appropriate substitutions [63]. Standardization and legislation concerning food products were also considered to maximize agreement between them and the positive FOPL (e.g., standards of whole-wheat flour, cereals, etc.).

#### 2.1.3. Consideration of Food Processing Level

High consumption of ultra-processed food is associated not only with higher rates of obesity and NCDs, but also autoimmune diseases and inflammatory bowel diseases [64,65,66]. The number of nutrients in food products does not necessarily reflect the processing level and neither does the calorie density of the product. Even the degree of food processing does not necessarily reflect the sugar, sodium, and saturated fat content of the foods. For example, 100% juice, sweetened fruit juices, and sugar-sweetened beverages, categorized as basic processed, moderately processed, and highly processed, respectively, contain almost identical amounts of sugar [67].

A significant dilemma was how to address the extensive use of food additives and their impact on the human body. There is substantial growth in the use of food additives, with more than 10,000 substances permitted for use in the USA [68]. Nonetheless, scientific evidence exists concerning the harmful effect on the health of some of them, although they are generally recognized as safe (GRAS), such as nitrates, nitrites, carrageenan, stabilizers, etc. There is specific concern regarding children, who might be at greater risk because of their higher exposure on a body-mass basis and immature body systems. The effect on the endocrine system is particularly worrying, especially in the early stages of life. In addition, there is insufficient information regarding the synergistic effects between food additives and the implications of cumulative exposure for years [69,70].

Therefore, the committee decided to profile the foods according to their processing level and not only based on their nutrients’ composition. Several systems for classification of food processing levels exist. Five of them were examined in a systematic review, which found the NOVA the most advantageous [71]. The NOVA classification system is based on the nature, extent, and purpose of food processing and consists of four groups: Unprocessed or minimally processed, processed culinary ingredients, processed, and ultra-processed foods and drinks [7,8]. The committee referred to the NOVA system as the basis for classification of foods, but has not fully adopted it. While in the NOVA system, the foods in the processed culinary ingredients’ category may contain food additives such as stabilizers and preservatives, the committee decided, in accordance with the principles of the Mediterranean diet, to allow positive labeling for foods with no food additives. Thus, for example, legumes are eligible for positive FOPL, but processed products containing protein extracts of legumes are not. Plain yogurt is eligible for positive FOPL, but yogurts with sweeteners are not.

#### 2.1.4. Consideration of the Natural Composition of the Foods

The criteria for the positive FOPL were defined according to the nutritional form of the raw food, without nutritional enrichment, to allow fresh foods to acquire a positive FOPL and not to encourage the industry to use food additives to meet the criteria. For example, the criteria for milk and dairy products were determined according to their natural content of fat, saturated fat, and calcium.

The criteria on which positive FOPL are based do not address the presence of pathogens, sensitivity to food, and ethical and religious elements, as done in other countries [17]. Reference to environmental aspects in the positive FOPL is a result of the nutritional guidelines that are based on the Mediterranean diet [56,57].

After the recommendations of the committee were presented to the MOH, they were published in the media and were sent to main stakeholders, for example, the civil society organizations and the Manufacturers’ Association. The MOH invited the public to comment on the proposed positive FOPL and offered two alternatives:

1. Enable mixtures between categories that include only grains, legumes, fruits, vegetables, and nuts.
2. Enable mixtures between all food groups while meeting each group’s criteria.

Public comments were gathered and an open meeting was held with the MOH representatives to discuss them. The meeting was attended by representatives of the committee, public health experts from a medical association and academia, civil society organizations, and the food industry.

Final modifications to the criteria were made during September–October 2019 while considering all comments. Most of the committee’s decisions were taken by consensus. In cases where unanimity could not be reached, the final decision was made according to the opinion of the majority of committee members.

### 2.2. Assessment of the Expected Prevalence of Positive FOPL

To assess the proportion of foods eligible for the positive FOPL and, hence, the visibility of the positive FOPL at the point of purchase, data from MABAT and BINAT were examined [13,14,62]. The eligible foods for positive and warning FOPL were determined based on their nutrient composition. The proportion of foods eligible for FOPL was calculated within all food groups and across different food groups. The data analysis was done before the reformulation process, carried out by the industry following the publication of the regulations of the warning FOPL and prior to their coming into force.

## 3. Results

### 3.1. The Criteria for Positive FOPL

The criteria for positive FOPL, as recommended by the committee, are summarized in Table 1. In general, the food products eligible for positive FOPL are foods in their natural form or with added spices or herbs or those that underwent minimal processing, with no food additives.

The criteria include a reference to the amount of sodium, fat, and saturated fat, where relevant, but not for energy or other nutrients. Generic principles, such as the amount of sodium per 100 g, were applied across all related food groups. Tailored criteria were set for specific food groups, such as the amount of fat and saturated fat in dairy products. Combinations of the categories, grains, legumes, fruits, vegetables, and nuts, were allowed, as long as the category-specific criteria were maintained. In addition, levels of sodium, saturated fat, and sugar should not exceed the values set for the warning FOPL (Appendix B).

In addition to the criteria for positive FOPL, the committee made further recommendations:

1. Encourage the labeling of unpackaged foods, such as vegetables, fruits, whole grains, and legumes to enhance the visibility of the positive FOPL and promote their consumption by the public (Appendix C).
2. Provide information about the criteria used for each food group (in addition to the prominent positive FOPL). If applicable, the recommended intake must be specified. Since the place on the food packages is limited, it has been suggested to publish the information on a dedicated Internet site.
3. Launch the positive FOPL together with a comprehensive campaign and dissemination of information concerning the Mediterranean diet recommendations.
4. Develop an evaluation plan for the public and industry acceptability of the positive FOPL. A measurement of changes in food purchasing and consumption behaviors (e.g., the degree of use of the positive FOPL by the retailers and the industry, the number of new products developed to meet the criteria, price changes, the public’s trust in the label, and health measures of the population).
5. Update the criteria for positive FOPL as needed. It has been suggested that the committee continues to meet every six months to review applications received from the public and the industry and assess the need to expand the criteria to include additional food products.

### 3.2. The Proportion of Foods Eligible for Positive FOPL

**Figure 1:**
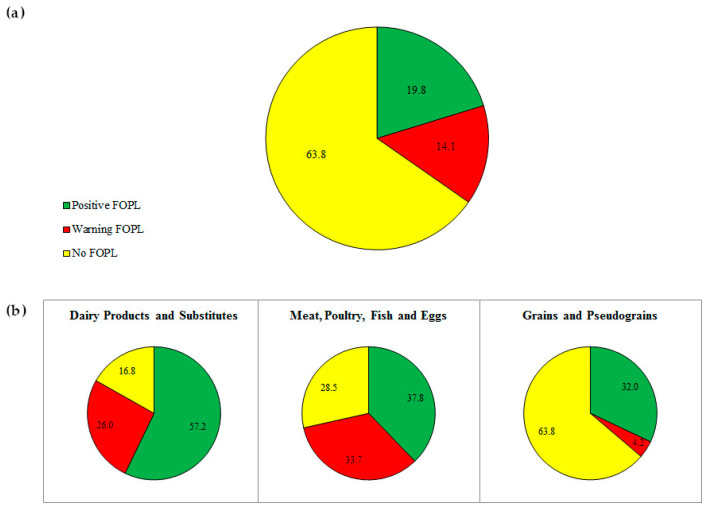
(**a**) The proportion (%) of food products from all food groups and (**b**) from selected food groups that would be eligible for positive FOPL. The green, red, and yellow areas represent the proportion of food within each group that is eligible for positive FOPL, warning FOPL, and no FOPL, respectively.

Based on the Israeli consumption data, 19.8% of food products were eligible for positive FOPL, and 14.1% of food products would carry warning FOPL (Figure 1a).

More than half (54%) of the food products eligible for positive FOPL were fruits and vegetables, 20% from the milk and dairy group, 14% from the grains group, 11% from the meat, poultry, fish and egg group, and 1% were legumes. Within the milk and dairy group, 57.2% of food products are eligible for positive FOPL. Within the meat, poultry, fish, and egg group and in the grains group, 37.8% and 32% of food products are eligible for positive FOPL, respectively (Figure 1b).

## 4. Discussion

This paper provides an overview of the process and professional considerations of the development of the Israeli positive FOPL. The established criteria were based on the new Israeli nutritional guidelines, focusing on the health advantages of the food and considering its processing level.

The Israeli unique approach for positive FOPL has a number of potential advantages. It provides clear and simple messages that should lead to healthier food purchases and eating choices across all population subgroups. This is particularly important since the prevalence of obesity in Israel is higher among the low socioeconomic class [1].

Positive FOPL, according to individual components (e.g., fibers or vitamins), might give the false impression of a healthy food product. This kind of label can create a health “halo” and affect the consumer’s judgment and change their perception of the overall healthfulness of the food product [72,73]. FOPL based on an algorithm or a score, like Nutri-Score, Traffic Light, and Health Star Rating, does not necessarily differentiate between recommended and less-recommended foods, for example, whole grains and refined grain foods [74]. Positive FOPL for the best product in a category can as well create a bias in the eyes of the public since being “the best among the worst” does not necessarily mean being healthy. Public trust can be negatively affected by the positive labeling of products perceived as not healthy [75].

Positive FOPL can cause consumers to pay less attention to the nutritional information detailed on the back of the package [76]. The public perceives FOPL as highly essential and relies on it. Therefore, it is of utmost importance that the positive FOPL will provide reliable information concerning the food products recommended for consumption [33].

Therefore, the committee decided to determine strict criteria, so that only food complying with the national nutritional guidelines will be eligible for positive FOPL and that ultra-processed foods would not be eligible for positive FOPL. The fact that the committee was independent and was not influenced by the food industry facilitated the establishment of these criteria.

A significant decision of the committee was that unpackaged food would be eligible for the positive endorsement label as well. This eliminates the negative impact of positive labeling of packaged foods only, which discriminates against unpackaged foods such as vegetables, fruits, whole grains, and legumes. On-shelf labeling of unpackaged food increases the exposure of the consumers to the positive FOPL at the point of purchase, increasing the extent of its implementation. This is particularly important in Israel, where most fruits and vegetables are sold in loose format, not only in open markets but also in retail stores and supermarkets. This model could fit well in low- and middle-income countries, where most food is sold in traditional retail environments.

An additional advantage is expected from reformulation of food products with no label in order to receive a positive FOPL, in parallel with the ongoing reformulation process already being done by the industry so as to avoid the warning labels.

The positive FOPL in Israel is free of charge. From past experience, public confidence has been compromised when food manufacturers had to pay a licensing fee to display the label on foods that met the criteria [75].

There are also several limitations of this FOPL system. The strict criteria limited the number of food categories and products eligible for positive FOPL. Although about 20% of food products are eligible for positive FOPL, more than half of them are fruits and vegetables. The result is that a small proportion of food will be positively labeled. Therefore, cooperation with the food retailers is needed in order to improve the visibility of the positive FOPL at the points of purchase.

As opposed to the mandatory warning FOPL, the positive FOPL in Israel is voluntary. The motivation to use the positive FOPL can stem from the interest of food companies and retailers to encourage buying, as a consumer is willing to pay for healthier foods [77]. In addition, the food companies and retailers can be perceived as responsible for their consumers’ health, which in turn may help build a positive image and a non-price competitive advantage [78,79]. In New Zealand and Australia, where labeling is voluntary, only 21% and 28% of the eligible food products were indeed labeled after four and three years, respectively [80,81]. The implementation of the positive FOPL by the industry must be evaluated. If it is not used voluntarily, a mandatory approach should be considered, as was concluded in Australia [81].

Concerns have been raised about the negative consequences of positive FOPL, such as higher food prices. However, experience in Australia indicates that healthier packaged food products were not consistently more expensive than less healthy products [82].

According to the WHO, the engagement of main stakeholders is important for the success of a FOPL system. Therefore, the recommendations of the committee were exposed for public comments before their final determination. The publication of the criteria has drawn criticism from the industry, for not including enough manufactured products. There was also criticism from the Israeli Forum for Sustainable Nutrition for permitting positive labeling on animal products. As opposed to this, fundamental support for the positive FOPL criteria was expressed by the Israeli Dietetic Association, The Israeli Association of Public Health Physicians, and the heads of the Schools of Public Health in Israel.

The positive FOPL can be used as a reference for several aspects of public health policy, e.g., food advertising for children, menu planning for preschools, schools, and other government-supervised frameworks, and subsidizing foods, in order to encourage their consumption by the entire population [83].

The clear and unequivocal criteria for the positive FOPL, and the fact that the industry was not involved in their determination, increase the likelihood of their being positively accepted and trusted by the public. Support from civil society organizations is vital to encourage the use of positive FOPL by the food companies and the consumers. A campaign advertising the positive FOPL together with the new nutritional recommendations can facilitate public understanding and use of the positive FOPL system [17,18,19,84].

In summary, this study suggests a unique methodology for the development of positive FOPL. It adds to the knowledge of existing FOPL systems and may guide other countries searching for comparative considerations during the food labeling process. 

An evaluation plan is needed to assess the degree of acceptance of the positive FOPL by industry, retailers, and the public, its impact on food consumption, and the long-term changes in diet-related public health. Periodical monitoring will allow for continuing improvements or adjustments of the criteria for the positive FOPL, as required.

## Figures and Tables

**Table 1:** Criteria for positive Front-of-package labeling (FOPL) for the different food groups.

Category	Subcategory	Allowed Additives ^1^	Maximum Levels per 100 g
Dairy products	Liquid milk	According to food standards	
	Fermented milk products	No additives except raw materials	Total fat ≤5%
	Cheeses	No additives except salt and spices ^2^	Total fat ≤5 g Sodium ≤200 mg
Soy products	Tofu	No additives except salt, spices and gelling agents, as allowed by food standards	Sodium ≤200 mg
	Soy Drink	Soybeans, water, salt	Sodium ≤50 mg
Vegetable oils	Avocado oil	No additives except tocopherols and ascorbic acid	
	Almond oil		
	Olive oil		
	Safflower oil		
	Sunflower oil		
Seeds, Nuts, Almonds	Raw or roasted/flour/spreads	No additives	
Grains and pseudo-grains	Whole grains ^3^ in any kind of preparation and packaging (e.g., roasted, cooked, baked, cut, steamed, frozen, vacuum-packed, tinned), not fried	No additives except salt and spices ^2^	Sodium ≤200 mg
	Flour from whole grains ground from the entire kernel		
	Puffed rice/crackers 100% whole grains	No additives except salt and spices	Sodium ≤200 mg
	Whole pasta according to food standards	No additives	
Legumes	Legumes whole kernel, in any kind of preparation and packaging (e.g., roasted, cooked, baked, cut, steamed, frozen, sprouted, vacuum-packed tinned), not fried	No additives except salt and spices	Sodium ≤200 mg
	Legume flour (100%)	No additives	
	Legume spreads (100%)	No additives except salt, spices, tahini, olive oil, lemon	Sodium ≤200 mg Total fat <7%
Tahini from whole sesame	Raw (not diluted)	No additives	
	Salad	No additives except salt, spices	Sodium ≤200 mg
Eggs	Fresh, unpeeled		
Fish	Fresh, chilled or frozen (raw), whole or parts	No additives	
	Baked, roasted or spiced	No additives except spices, salt	Sodium ≤200 mg
	Canned	No additives except spices, oils from the list above, salt, water	Sodium ≤200 mg
Poultry	Fresh, chilled or frozen (raw), whole or parts	No additives	Sodium ≤300 mg
	Baked, roasted or spiced	No additives except spices, salt	Sodium ≤200 mg
Fruits	All kinds of preparation and packaging (e.g., fresh, frozen, cooked, pureed, pickled, roasted, baked, vacuum-packed, tinned). Not jams, confitures, not fried, no juice, not powder, not dried/dry except reconstituted from freeze-drying	No additives	
Vegetables	All kind of preparation and packaging (e.g., fresh, frozen, cooked, pureed, pickled, roasted, baked, vacuum-packed, tinned), not fried	No additives except spices, salt	Sodium ≤ 200 mg
Tea	Tea and herbal infusion according to food standards	No additives	

^1^ Additives: Any additives including food additives as defined by Israeli law except water for plant-based foods. ^2^ Spice (or herb): As defined in Standard 1359: Mixed spices, powders, and other mixes for adding flavor to food (103.1). A plant or its parts (such as roots, bulbs, shoots, leaves, seeds) whole or cut up or ground, which are added to food to give taste or smell. These are not flavoring powders, spice mixes, (103.2), powders, or mixes that contain spice extracts. Spices/herbs in the cheese category should meet the definition of spices/herbs in this document and include garlic, onion, dill, parsley. ^3^ Whole grains: Containing the entire kernel, ground, cracked, or flaked with its main anatomical parts, endosperm, germ, and bran, being in the same relative amounts as found in the entire caryopsis. The term “whole grain” also includes flour from the whole grain (Israel Standard 1241, paragraph 1.3.4) and pseudo-grains as defined by the American Association for Clinical Chemistry (AACC) and the Food and Drug Administration (FDA), e.g., quinoa, buckwheat, and teff.

**Figure A1:**
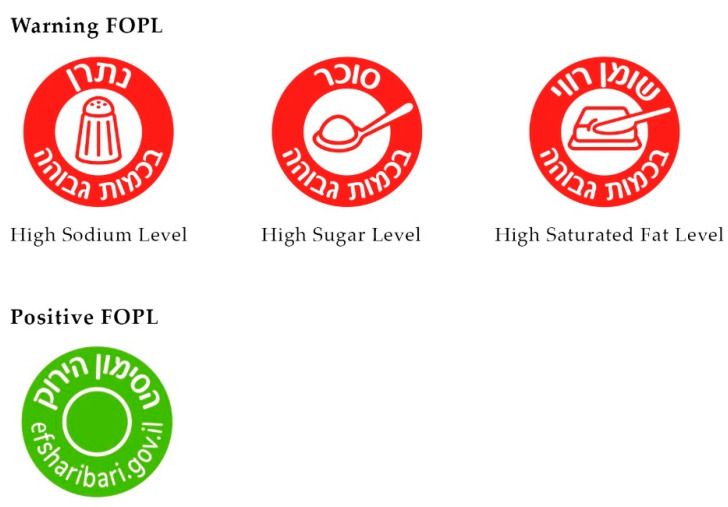
Warning (red) and positive (green) FOPL graphics.

**Figure A2:**
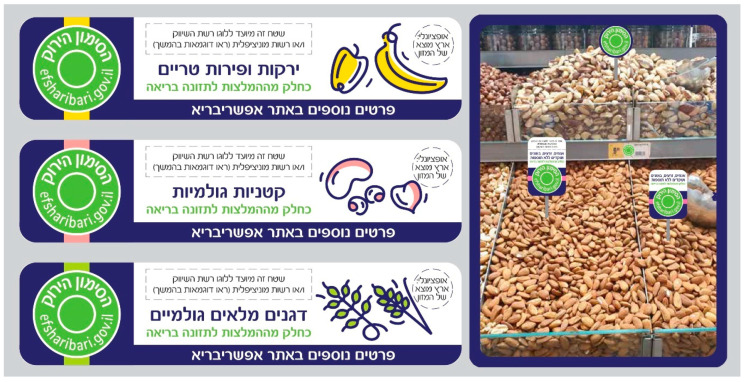
Israeli Ministry of Health proposal for labeling unpackaged foods at the point of purchase.

**Figure A3:**
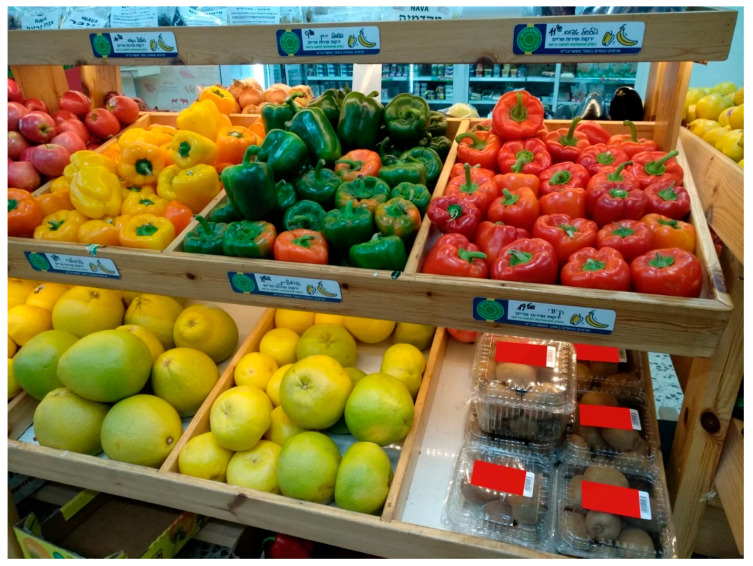
Labeling unpackaged fruits and vegetables in a grocery shop.

## Appendix B

**Table A1:** Maximum levels of sodium, sugars, and saturated fatty acid, which, if exceeded, will require red labeling on the front of the package for either solid food or liquid food.

Nutrient	Amount in 100 g Solid Food	Amount in 100 mL Liquid Food
**First stage, January 2020**		
Sodium (mg)	500	400
Total sugars (g)	13.5	5
Total saturated fatty acids (g)	5	3
**Second stage, January 2021**		
Sodium (mg)	400	300
Total sugars (g)	10	5
Total saturated fatty acids (g)	4	3
